# Persistent SARS‐CoV‐2 RNA Positivity in a Chronic Lymphocytic Leukaemia Patient for 127 Days After Disease Onset: A Case Report

**DOI:** 10.1002/rcr2.70767

**Published:** 2026-10-05

**Authors:** Farnoosh Khamseh, Amir‐Hossein Moghtader‐Mojdehi, Zahra Nemati, Mehrdad Haghighi

**Affiliations:** ^1^ Department of Infectious Diseases and Tropical Medicine, Imam Hossein Hospital, School of Medicine Shahid Beheshti University of Medical Sciences Tehran Iran; ^2^ Department of Critical Care Nursing, School of Nursing and Midwifery Tehran University of Medical Sciences Tehran Iran

**Keywords:** chronic lymphocytic leukaemia, COVID‐19, immunocompromised host, persistent viral shedding, SARS‐CoV‐2

## Abstract

Chronic lymphocytic leukaemia (CLL) is characterised by profound immune dysfunction, increasing susceptibility to severe and persistent infections such as COVID‐19. Our case report presents a 69‐year‐old woman with CLL who exhibited a prolonged duration of SARS‐CoV‐2 RT‐PCR positivity—127 days—despite receiving standard therapies including dexamethasone and remdesivir. The patient, previously on maintenance ibrutinib, presented with fever and ground‐glass opacities on CT scan and laboratory findings revealed marked lymphocytosis, elevated inflammatory markers and hepatic and renal dysfunction. Persistent viral shedding was attributed to CLL‐associated hypogammaglobulinemia and chemotherapy‐induced immunosuppression, resulting in impaired antibody response and eventual fatal hypoxemia. This case underscores the challenges of managing COVID‐19 in CLL patients and highlights the need for individualised treatment strategies, including consideration of monoclonal antibodies and immunoglobulin replacement, to address prolonged viral replication and improve clinical outcomes in this vulnerable population.

## Graphical abstract

This case report presents a 69‐year‐old woman with chronic lymphocytic leukaemia who exhibited prolonged SARS‐CoV‐2 RNA positivity for 127 days despite standard treatments. This case emphasises the profound immunodeficiency in CLL patients and highlights the necessity for tailored therapeutic strategies to manage persistent COVID‐19 infections.

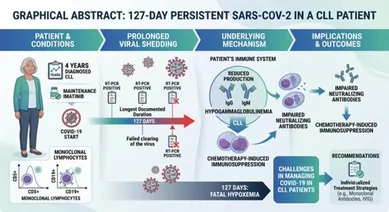

## 1 Introduction

Chronic lymphocytic leukaemia (CLL) is characterised by the progressive accumulation of monoclonal lymphocytes with a distinctive immunophenotype (i.e., CD5^+^, CD19^+^, CD20^dim^, CD23^+^ and SmIg^dim^) in peripheral blood, bone marrow and lymphoid tissues. Patients with CLL frequently present with immune disturbances and infections, which constitute a notable feature of the disease compared to other chronic lymphoproliferative disorders [1]. Patients with haematological malignancies, especially chronic lymphocytic leukaemia (CLL) are often on therapeutic drugs that suppress the immune system, putting them at a particularly high risk for persistent viral replication or severe secondary infections when infected with SARS‐CoV‐2 [2]. In this study, we present a CLL patient with an unusually prolonged duration of RT‐PCR positivity lasting 127 days.

## 2 Case Report

A 69‐year‐old woman, diagnosed with CLL 4 years ago, presented with a fever. The patient had previously been diagnosed with CLL and had been receiving ibrutinib as maintenance therapy for 2 years prior to the onset of her COVID‐19 symptoms.

CT‐scan revealed a ground‐glass opacity (GGO) involvement pattern. RT‐PCR confirmed SARS‐CoV‐2 infection. Two weeks after clinical recovery, RT‐PCR remained positive. Twenty‐eight days later, the patient again presented with fever and weakness. The patient was initially asked to cease ibrutinib therapy upon admission, a common practice recommended for patients diagnosed with COVID‐19 to minimise potential drug‐related complications and immunosuppression. In this case, dexamethasone was administered to manage inflammation and remdesivir was used as an antiviral therapy, standard treatments for hospitalised COVID‐19 patients aiming to reduce viral replication and mitigate cytokine storm effects.

Despite treatment, RT‐PCR tests continued to yield positive results. Serial RT‐PCR tests for SARS‐CoV‐2 were performed using nasopharyngeal aspirates, and viral genome detection persisted up to 127 days after symptom onset. The patient remained under quarantine and home observation. Eventually, after 127 days, she experienced a decrease in oxygen saturation and passed away in the intensive care unit. Table 1 presents the important laboratory findings of the patient (Table 1).

**TABLE 1:** Laboratory findings of the CLL patient with COVID‐19.

Variable (unit)	Finding
Blood WBC (× 10^9^/L)	50
Lymphocyte (%)	88%
Neutrophil (%)	21%
CRP (mg/L)	200
ESR (mm/Hour)	119
Alb (mg/L)	3.5
Total protein (mg/L)	4.7
ALT (U/L)	87
AST (U/L)	75
Alp (U/L)	225
Cr (mg/dl)	1.5
BUN (mg/dl)	40

Abbreviations: Alb, albumin; Alp, alkaline phosphatase; ALT, alanine aminotransferase; AST, aspartate aminotransferase; BUN, blood urea nitrogen; Cr, creatinine; CRP, C‐reactive protein; ESR, erythrocyte sedimentation rate; WBC, absolute count of white blood cells.

## 3 Discussion

This study highlights a CLL patient with a persistent and prolonged duration of SARS‐CoV‐2 RNA positivity, lasting 127 days after symptom onset. As early reports from China indicated, individuals with cancer are more susceptible to SARS‐CoV‐2, although initial data on the prevalence and features of COVID‐19 in different cancers, including CLL, were limited. This extended period of viral shedding can be attributed to the profound immunodeficiency associated with CLL, further compromised by ongoing chemotherapy and ibrutinib therapy [2]. Specifically, CLL patients often exhibit hypogammaglobulinemia, characterised by reduced levels of protective antibodies such as IgG, IgA and IgM, which are crucial for neutralising SARS‐CoV‐2 and preventing viral dissemination [1]. This humoral immune deficiency impairs the development of effective antibodies against SARS‐CoV‐2, leading to prolonged viral replication and shedding. Our patient's lack of seroconversion underscores this phenomenon, aligning with studies indicating that CLL patients exhibit suboptimal antibody responses to SARS‐CoV‐2 infection [1].

The high mortality rate observed in CLL patients with COVID‐19 is intricately linked to their compromised immune status and pre‐existing comorbidities. Our patient's advanced age, combined with ongoing chemotherapy, significantly elevated the risk of severe outcomes. Other studies have similarly shown that CLL patients are more susceptible to experiencing severe COVID‐19, exhibiting higher rates of hospitalisation, intensive care unit admissions and mortality compared to the general population. Similar instances of extended SARS‐CoV‐2 positivity have been documented in other immunocompromised individuals [1]. While treatments such as remdesivir and dexamethasone are standard in managing COVID‐19, they may not suffice in achieving viral clearance among CLL patients, as evidenced by our case [3]. Although prolonged viral shedding has been documented in various immunocompromised states, a pivotal distinguishing feature of our case is demonstrating that the early cessation of targeted therapy, combined with standard antiviral and corticosteroid administration, was entirely insufficient to halt continuous viral replication, ultimately leading to fatal hypoxemia by Day 127. In contrast, the utilisation of monoclonal antibodies like casirivimab/imdevimab has shown promise in facilitating rapid viral clearance in CLL patients grappling with persistent infections, thus highlighting a potential therapeutic avenue [4]. This underscores the critical importance of implementing effective treatment strategies to mitigate prolonged viral replication and minimise potential transmission risks. Addressing these comorbidities through targeted therapies, such as intravenous immunoglobulin (IVIG) infusions, or supportive care measures may hold promise in improving outcomes among CLL patients infected with COVID‐19.

This case underscores a CLL patient's unprecedented 127‐day SARS‐CoV‐2 RNA positivity, stemming from profound immunodeficiency and impaired antibody response, reinforcing the heightened susceptibility of CLL patients to severe COVID‐19. The prevalence, clinical characteristics and outcomes of CLL patients with COVID‐19 infection have yet to be fully established, though preliminary data from large patient series can be valuable for understanding and managing such cases, like in this report. This highlights the need for tailored strategies beyond standard treatments like remdesivir and dexamethasone, with monoclonal antibodies showing promise, especially given the high mortality linked to comorbidities. Furthermore, addressing hypogammaglobulinemia and preventing viral variants requires vigilance, suggesting interventions like IVIG and robust monitoring. Overall, future research should prioritise personalised approaches to optimise outcomes for CLL patients with COVID‐19.

## Author Contributions

**Farnoosh Khamseh:** writing – original draft, writing – review and editing, conceptualization, methodology. **Amir‐Hossein Moghtader‐Mojdehi:** investigation, validation. **Zahra Nemati:** data curation, writing – original draft, visualization. **Mehrdad Haghighi:** writing – review and editing, methodology, project administration.

## Funding

This research received no specific grant from any funding agency in the public, commercial or not‐for‐profit sectors.

## Consent

The authors declare that written informed consent was obtained for the publication of this manuscript and accompanying images using the consent form provided by the Journal.

## Conflicts of Interest

The authors declare no conflicts of interest.

## Data Availability

The data that support the findings of this study are available from the corresponding author upon reasonable request.
